# Incidence and outcomes of vasa praevia in the United Kingdom

**DOI:** 10.3310/nihropenres.13696.2

**Published:** 2024-12-05

**Authors:** George Attilakos, Anna L David, Ruth Tunn, Marian Knight, Peter Brocklehurst

**Affiliations:** 1 Fetal Medicine Unit, University College London Hospitals NHS Foundation Trust, 235 Euston Road, London, NW1 2BU, UK; 2 Elizabeth Garrett Anderson Institute for Women’s Health, University College London, Medical School Building, Huntley Street, London, WC1E 6AU, UK; 3 NIHR University College London Hospitals Biomedical Research Centre, 149 Tottenham Court Road, London, W1T 7DN, UK; 4 National Perinatal Epidemiology Unit, University of Oxford, Old Road Campus, Headington, Oxford, OX3 7LF, UK; 5 Birmingham Clinical Trials Unit, Institute of Applied Health Research, University of Birmingham, Room 106, Public Health Building, Edgbaston, Birmingham, B15 2TT, UK

**Keywords:** Keywords: Vasa praevia, incidence, outcomes, perinatal mortality, velamentous cord insertion, pregnancy, surveillance, UK Obstetric Surveillance System

## Abstract

**Background:**

Vasa praevia is an obstetric condition in which the fetal vessels run through the membrane over the internal cervical os, unprotected by the placenta or umbilical cord. It is associated with perinatal mortality if not diagnosed antenatally. We estimated the incidence and investigated outcomes of vasa praevia in the UK.

**Methods:**

We conducted a population-based descriptive study using the UK Obstetric Surveillance System (UKOSS). Cases were identified prospectively through monthly UKOSS submissions from all UK hospitals with obstetrician-led maternity units. All women diagnosed with vasa praevia who gave birth between 1st December 2014 and 30th November 2015 were included. The main outcome was estimated incidence of vasa praevia with 95% confidence intervals, using 2015 maternities as the denominator.

**Results:**

Fifty-one women met the case definition. The estimated incidence of diagnosed vasa praevia was 6.64 per 100,000 maternities (95% CI 5.05-8.73). Of 198 units, 10 (5%) had a vasa praevia screening programme; one of these 10 units identified 25% of the antenatally diagnosed cases. Among women who had vasa praevia diagnosed or suspected antenatally (n=28, 55%), there were no perinatal deaths or hypoxic ischaemic encephalopathy (HIE). Twenty-four women with antenatal diagnosis were hospitalised at a median 32 weeks’ gestation and caesarean section was scheduled at a median 36 weeks’ gestation. When vasa praevia was diagnosed peripartum (n=23, 45%), the perinatal mortality rate was 37.5% and 47% of survivors developed HIE.

**Conclusions:**

The incidence of diagnosed vasa praevia was lower than anticipated. There was high perinatal mortality and morbidity for cases not diagnosed antenatally. The incidence of antenatally identified cases was much higher in the few centres that actively screened for this condition, and the perinatal outcomes were better. However, this group were all delivered by caesarean section and may include women who would not have experienced any adverse perinatal outcome.

## Plain Language Summary

Vasa praevia is a pregnancy complication in which the blood vessels that connect the mother and fetus run across the opening of the womb, without protection from the placenta or umbilical cord. During birth, the vessels can tear. This can result in rapid blood loss from the baby and in some cases, death of the baby. We investigated how common vasa praevia is in the UK, and how women with the condition and their babies fared.

The UK Obstetric Surveillance System (UKOSS) collects anonymous information from all maternity units in the UK about pregnant women who have certain medical conditions. UKOSS reporters provided information about all women with vasa praevia who gave birth between December 2014 and November 2015. We identified 51 women with vasa praevia, meaning vasa praevia was diagnosed less often in the UK than we had expected based on studies from other countries. Twenty-eight women were diagnosed during the antenatal period, while 23 were diagnosed during labour or after giving birth. Pregnant women in the UK are not screened for vasa praevia as standard, and some women may have had vasa praevia that was not diagnosed. A small number (5%) of maternity units in our study did offer screening for vasa praevia in their pregnant population. One of these units identified a quarter of all the women who had vasa praevia diagnosed during pregnancy.

Babies born to women whose vasa praevia was diagnosed during pregnancy had good outcomes. All of these women gave birth by planned caesarean section, and they and their babies survived. Babies born to women whose vasa praevia was suspected or diagnosed during labour or after birth had worse outcomes. Around 40% were stillborn or died shortly after birth, and about half of those who survived had brain damage caused by lack of oxygen.

## Introduction

Vasa praevia is a rare obstetric complication which is defined as fetal vessels coursing through the membranes over the internal cervical os and below the fetal presenting part, unprotected by placental tissue or the umbilical cord ^1^. This can be secondary to a velamentous cord insertion (vasa praevia type 1) or to fetal vessels running between lobes of a placenta with one or more accessory lobes (vasa praevia type 2) ^1^. A third type, first described in 2019, involves vessels that leave then re-enter the placenta in a “boomerang” path (vasa praevia type 3) ^2^. Rupture of the vessels during labour can lead to rapid fetal exsanguination and fetal death.

Vasa praevia can be associated with high perinatal mortality if it is not diagnosed antenatally ^3, 4^. Although antenatal screening is possible, a suspicion of vasa praevia usually leads to caesarean section before labour, avoiding the chance of fetal vessel rupture during birth. However, the rarity of the condition; the possibly high false positive rate of antenatal diagnosis; the potential harms of iatrogenic late preterm delivery, such as neurodevelopmental issues ^5^; and the inability of antenatal diagnosis to predict which cases of vasa praevia will rupture and bleed during labour means that antenatal screening has the potential for greater harm than benefit. Consequently, routine screening for vasa praevia is not advised by Royal College of Obstetricians and Gynaecologists guidelines ^6^ or the National Screening Committee (NSC) ^7, 8^, although both organisations indicate a paucity of evidence on which to base a recommendation. Conversely, a recent international expert consensus statement supported routine screening because of the potential reduction in preventable perinatal mortality ^9^.

A recent systematic review of 24 studies suggested a mean incidence of vasa praevia of 7.9 (95% CI 5.9–10.1) per 10,000 pregnancies ^10^. The majority of the included studies were retrospective, single-centre investigations and ultrasound protocols for antenatal detection of vasa praevia varied between studies. Two included prospective studies were small (4 and 11 cases of vasa praevia) and each based in two institutions ^11, 12^. Two large population-based studies were identified: a retrospective study of the California birth cohort from 2007 to 2012 found an incidence of 2 per 10,000 live singleton births ^13^, while a prospective study using the Australasian Maternity Outcomes Surveillance System (AMOSS) showed a similar incidence of 2.1 per 10,000 women giving birth ^1^.

Data from the UK are limited; a retrospective study of data from a prospective vasa praevia screening programme in a single UK fetal medicine unit estimated an incidence of 8 per 10,000 singleton pregnancies, in a study population of 26,830 ^14^, while a 5-year historical cohort study at a single UK hospital with a screening program estimated a similar incidence of 7.7 per 10,000 births ^15^. However, no study has investigated the population incidence of vasa praevia in the UK prospectively or using nationwide data. We used the United Kingdom Obstetric Surveillance System (UKOSS) to prospectively estimate the incidence and investigate outcomes of vasa praevia in the UK. We also surveyed all UKOSS reporting centres about vasa praevia antenatal screening practices.

## Methods

### Patient and Public Involvement

Patients were not directly involved in the design of the study. Two members of the public were indirectly involved in the design of the study via representatives on the UKOSS Steering Committee, which reviews, comments on, and approves all studies to be run through UKOSS.

This is a population-based descriptive study using the UK Obstetric Surveillance System (UKOSS). UKOSS was established to study rare pregnancy disorders through routine monthly reporting from all UK maternity units. The UKOSS methodology has been previously described ^16^. In summary, nominated reporting clinicians (midwives, obstetricians and/or obstetric anaesthetists) in each hospital were sent a card each month, which included a simple tick-box to indicate a case of vasa praevia (along with other conditions studied). When a case was reported, a data collection form was sent to the reporting clinician to collect details on demographics, pregnancy risk factors, ultrasound diagnosis, antenatal and intrapartum management, delivery and neonatal outcome. We asked about the following risk factors for vasa praevia: IVF conception, low lying placenta, marginal or velamentous cord insertion, and bilobed or succenturiate lobed placentation. The full data collection form for this study can be found here: https://www.npeu.ox.ac.uk/assets/downloads/ukoss/forms/UKOSS-Vasa-Praevia-V1.pdf.

We used a robust case definition where each case was required to meet at least one clinical criterion and at least one postnatal confirmation criterion ( Table 1). Cases were confirmed against the case definition. The woman’s year of birth, hospital and estimated date of delivery were used to exclude duplicate cases.

**Table 1.** Case definition.

Clinical criteria (at least one of)	Confirmation criteria (at least one of)
Suspected VP on antenatal ultrasound ≥18 weeks gestation, and confirmed on antenatal ultrasound ≥31 weeks gestation (if not delivered prior to 31 weeks)	Clinical examination of the placenta confirming intact or ruptured velamentous vessels. These may be a velamentous insertion of the umbilical cord or exposed fetal vessels between placental lobes
Palpation or visualisation of the fetal vessels during labour	Confirmation of VP on pathological examination of the placenta
Rupture of membranes with bleeding associated with fetal death/ exsanguination or severe neonatal anaemia	Torn umbilical cord or placenta (not able to provide placental examination)
Antenatal or intrapartum bleeding of fetal origin with pathological CTG and/or positive Apt test	
VP documented in medical records as reason for admission and caesarean section	

VP: vasa praevia; CTG: cardiotocography

We collected data for vasa praevia cases in births occurring between 1 ^st^ December 2014 and 30 ^th^ November 2015. To establish the existence of screening programmes for vasa praevia, we asked each reporting centre the following question: “Between the 1st of December 2014 and the 30th of November 2015 was there a formal screening programme for vasa praevia at your hospital/centre?”.

Estimated incidence with 95% confidence intervals was calculated using the number of maternities for 2015 as denominator, using published data from the Office for National Statistics (England and Wales) ^17^, National Records of Scotland ^18^ and the Northern Ireland Statistics and Research Agency ^19^. For the purpose of the analyses, those cases diagnosed or suspected in the antenatal period were labelled as “antenatal”. Those cases not suspected/diagnosed in the antenatal period, but identified either during labour/delivery or after birth we labelled as “peripartum”.

We used chi-square test, Fisher exact test, t-test and Mann-Whitney U tests as appropriate for statistical comparisons. We used SPSS 22.0 (IBM Corp., Armonk, NY, USA) for statistical analyses.

### Ethics

The UK Obstetric Surveillance System general methodology was approved by the London Multi-Centre Research Ethics Committee (04/ MRE02/45; 24 September 2004) and this study was approved by the Proportionate Review Sub-committee of the East Midlands-Derby NRES Committee (14/EM/1237; 10 November 2014). Consent was not required for the collection of anonymous routine data.

## Results

**Figure 1.**
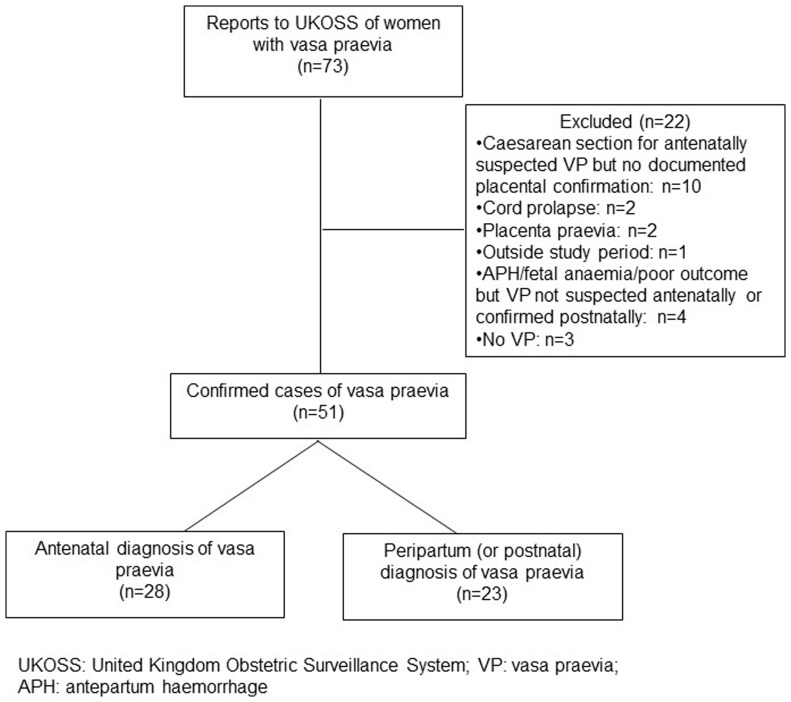
Reported and confirmed cases of vasa praevia during the study period.

Following exclusion of duplicates, UKOSS received reports of 73 women with vasa praevia. However, only 51 met the case definition (details of excluded reports are in Figure 1). Most excluded reports did not meet the placental confirmation criteria of our case definition: 10 had planned caesarean section for suspected vasa praevia but there was no documentation of the placenta being examined or sent to histopathology after birth, so there was no confirmation of vasa praevia; 7 did not have vasa praevia (2 had cord prolapse, 2 had placenta praevia, 3 others had suspected vasa praevia but no evidence of vasa praevia on postnatal examination of the placenta and membranes); and for 4 others, vasa praevia was not confirmed on histopathology despite suspicious delivery events or outcomes.

The total number of maternities for 2015 was 768,161. Therefore, we calculated the estimated incidence of vasa praevia in the UK as 6.64 per 100,000 maternities (95% CI 5.05–8.73). This equates to approximately 1 case for every 15,062 maternities.

Of the 51 cases included, 28 cases (55%) were antenatal (diagnosed or suspected in the antenatal period). The remaining 23 cases (45%) were peripartum (not suspected/diagnosed in the antenatal period). All but one of these 23 cases were associated with bleeding during labour or during induction of labour.

Demographic data and pregnancy characteristics are summarised in Table 2. A low placenta or a succenturiate lobe were more likely to have been identified in the antenatally diagnosed cases. Otherwise, there were no differences between the two groups. It is notable that all cases had at least one risk factor for vasa praevia.

**Table 2.** Maternal characteristics of vasa praevia cases.

Characteristics	Total (n=51)	Antenatal (n=28)	Peripartum (n=23)	p *
**Maternal age (years)**	32.7 (4.5)	33.3 (4.4)	31.9 (4.7)	0.29
**Body Mass Index**	25.2 (4.5)	24.4 (3.3)	26.0 (5.7)	0.22
**Ethnic group**				0.27
***Any white background***	41 (80)	20 (71)	21 (91)	
***Other***	10 (20)	8 (29)	2 (9)	
**Nulliparous**	25 (49)	16 (57)	9 (39)	0.34
**Smoking during pregnancy**	7 (14)	3 (11)	4 (17)	0.25
**Risk factors for VP**				
***Low lying placenta***	34 (67)	24 (86)	10 (43)	0.001
***Velamentous cord insertion***	25 (49)	15 (54)	10 (43)	0.26
***Bilobed placenta***	5 (10)	2 (7)	3 (13)	0.75
***Succenturiate lobe***	13 (25)	10 (36)	3 (13)	0.07
***Marginal cord insertion***	3 (6)	1 (4)	2 (9)	0.21
***IVF conception***	8 (16)	6 (21)	2 (9)	0.26
***At least one risk factor***	51 (100)	28 (100)	23 (100)	

Data are presented as mean (SD) or n (%).

* P-value for comparison between antenatal and peripartum diagnosis cases.

The clinical presentation and management are summarised in Table 3. Of the 11 women in the peripartum diagnosis group admitted to hospital antenatally, three were admitted for reasons relating to the position or movement of the fetus; four for maternal indications unrelated to vasa praevia, such as elevated blood pressure; and two for antepartum haemorrhage that progressed to birth. Notably, two women were admitted for antepartum bleeding earlier in the pregnancy, which could indicate a missed opportunity for vasa praevia diagnosis.

**Table 3.** Clinical management and presentation.

	Antenatal diagnosis n=28	Peripartum diagnosis n=23	p
**Number of scans after 17 weeks of** **gestation**			0.03
***1***	0 (0)	4 (17)	
***2***	2 (7)	5 (22)	
***3***	5 (18)	10 (43)	
***4***	10 (36)	2 (9)	
***5 or more***	11 (39)	2 (9)	
**Antenatal bleeding**	0 (0)	10 (43)	<0.0001
**Bleeding during labour**	0 (0)	8 (35)	0.002
**Bleeding at membrane rupture**	0 (0)	13 (57)	<0.0001
**Antenatal hospital admission**	24 (86)	11 (48)	0.006
**Cervical length measured**	10 (36)	0 (0)	0.001
**Cervical length used in decision to admit**	3 (11)	0 (0)	0.006
**Fetal fibronectin testing**	1 (4)	0 (0)	
**Fetal fibronectin used in decision to admit**	0 (0)	0 (0)	
**Antenatal steroids**	25 (89)	2 (9)	<0.0001
**CTG classification (where used)**			<0.0001
***Normal***	7 (25)	4 (17)	
***Suspicious***	0 (0)	3 (13)	
***Pathological***	1 (4)	11 (48)	
**Delivery by CS**	28 (100)	22 (96)	0.45
**Planned CS**	28 (100)	3 (13)	<0.0001
**Gestation of planned CS**	36.7 (34.2–39.0)	37.7 (37.7–37.7) (1 case)	1.000
**Gestation at delivery**	36.4 (31.3–39.6)	38 (36.1 – 39.9)	<0.0001
Urgency category *			<0.0001
***Category 1***	0 (0)	19 (83)	
***Category 2***	6 (21)	1 (4)	
***Category 3***	3 (11)	2 (9)	
***Category 4***	19 (68)	0 (0)	
**Anaesthesia method**			
***Regional***	25 (89)	7 (30)	<0.0001
***General***	3 (11)	15 (65)	<0.0001
**PPH > 1000 ml**	3 (11)	1 (4)	0.38
**Placenta examined**	28 (100)	21 (91)	0.28
**Placenta to pathology**	9 (32)	15 (65)	0.03

Data are presented as n (%) or median (range). *Urgency classification: 1- Immediate threat to life of woman or fetus; 2 – Maternal or fetal compromise which is not immediately life threatening; 3 – needing early delivery but no maternal or fetal compromise; 4 – At a time to suit the woman and maternity team. CTG: cardiotocography; CS, caesarean section; PPH, postpartum haemorrhage

Most peripartum-diagnosed cases presented with bleeding and this bleeding was at membrane rupture for 13 (57%) of these cases. Of the 23 women diagnosed peripartum, 22 (96%) delivered by caesarean section, of which 83% were category 1 surgeries; 15 women (65%) had general anaesthesia. There was one vaginal birth of a stillborn baby in this group. Of the 23 peripartum-diagnosed cases, 20 (87%) were born after 37 weeks and all after 36 weeks. 

Only 14% (n=4) of antenatally diagnosed cases were not admitted to hospital prior to delivery. The cervical length was measured in 10 (36%) of the antenatally diagnosed cases but it was used as a factor in the decision to admit for only 3 (11%) cases. Fetal fibronectin was measured in only one case and it was not a factor in the decision to admit. The median gestation at diagnosis was 29 weeks, with the earliest at 17 and the latest at 36 weeks. The earliest admission for antenatally diagnosed vasa praevia was at 21 weeks. This woman had 4 admissions for a total of 73 days. Only 3 other women with an antenatal diagnosis of vasa praevia were admitted to hospital prior to 30 weeks (two of them because of vaginal bleeding). The median gestation at admission was 32.6 weeks with the latest admission at 36.5 weeks. The median duration of admission was 6 days (range 1–73). Seventeen (61%) women with antenatal diagnosis stayed in hospital for more than 10 days. The 4 women who were not admitted had planned caesarean sections at 38–39 weeks.

All antenatally diagnosed cases were scheduled for a caesarean section, with the majority scheduled between 36 and 37 weeks. The earliest scheduled caesarean section was at 34 weeks (n=4) and the latest at 39 weeks (n=2). However, 9 (32%) of these women had a category 2 or 3 caesarean section earlier than planned. The earliest caesarean birth was at 31 weeks (n=1) because of recurrent antepartum bleeding. Only one other woman was delivered before 34 weeks, because of threatened preterm labour. Of the prenatally diagnosed cases, 25 (89%) had regional anaesthesia.

The perinatal outcomes are summarised in Table 4. There was a multiple pregnancy in the peripartum diagnosis group, so the outcomes refer to 52 fetuses. There were 4 stillbirths and 5 neonatal deaths in the peripartum group, making the perinatal mortality rate 37.5% for this group. The overall perinatal mortality rate was 17%. Almost 50% (7 of 15) of the surviving babies in the peripartum group had hypoxic ischaemic encephalopathy (HIE). In the group with peripartum diagnosis, 11 (46%) of babies had a blood transfusion for anaemia. There were no stillbirths, neonatal deaths or HIE cases in the antenatally diagnosed group but 2 babies in this group developed respiratory distress syndrome after delivery at 35–36 weeks.

**Table 4.** Perinatal outcomes.

	Antenatal diagnosis (n=28)	Peripartum diagnosis (n=24 *)	p
**Gender**			0.58
***Male***	13 (46)	13 (54)	
***Female***	15 (54)	11 (46)	
**Birthweight (g)**	2685 (±488)	3126 (±378)	0.001
**Stillbirth**	0 (0)	4 (17)	0.04
**Neonatal death**	0 (0)	5 (21)	0.002
**NICU admission**	10 (36)	15 (62)	0.006
**Anaemia**	0 (0)	9 (37)	<0.0001
**Blood transfusion**	0 (0)	11 (46)	<0.0001
**Hypoxic ischaemic** **encephalopathy**	0 (0)	7 (29)	0.002
**Seizures**	0 (0)	6 (25)	0.007
**Renal failure**	0 (0)	4 (17)	0.04

Presented as mean (±SD) or n (%). NICU: neonatal intensive care unit

* There was a multiple pregnancy in the peripartum diagnosis group, so the outcomes refer to 52 fetuses in total

 Of the 198 reporting centres, 174 (88%) replied to our question: “Between the 1st of December 2014 and the 30th of November 2015 was there a formal screening programme for vasa praevia at your hospital/centre?”. Only 10 hospitals (6% of respondents) declared they had a formal screening programme for vasa praevia during the time of the study. Fifteen replies (9%) were “unknown”. One of the 10 hospitals with screening programmes reported 7 cases, all antenatally diagnosed (14% of all cases and 25% of all antenatally diagnosed cases). The other 9 hospitals did not report any cases. This difference in antenatally diagnosed cases between screening and non-screening hospitals was statistically significant ( Table 5).

**Table 5.** Cases diagnosed in centres with and without a formal antenatal screening program (n=51).

	Number of cases identified	Perinatal deaths	Denominator numbers of births	Estimated incidence (95% CI) per 100,000 births *	Perinatal mortality (95% CI) per 100,000 **
**With screening** **programme**	7	0	42,814	16.4 (6.6–33.7)	0 (0–8.6)
**Without screening** **programme (or** **not known)**	44	9	739,906	6.0 (4.3–8.0)	1.2 (0.6–2.3)

Birth data from MBRRACE-UK perinatal mortality report

* p=0.021 when comparing screened to unscreened (Fisher’s exact test)

** p=1.0 when comparing screened to unscreened (Fisher’s exact test) CI: confidence interval

## Discussion

This prospective population-based study estimated a lower than anticipated incidence of vasa praevia in the UK: 6.64 per 100,000 maternities, or 1 case for every 15,062 maternities. There was high perinatal mortality and morbidity with peripartum diagnosis. The estimated incidence of antenatally identified cases in the few centres that actively screened for this condition was much higher, and the perinatal outcomes were better. However, this group were all delivered by caesarean section and some infants had respiratory morbidity as a consequence of elective preterm delivery.

A significant strength of our study is its prospective population-based design, which does not rely on routinely coded data to ascertain cases. This is a national study, conducted at all obstetric units in the UK and therefore covering the whole of the pregnant population. UKOSS is a well-established and validated system for identifying cases of uncommon pregnancy complications and, because it covers all obstetric units, is not subject to the usual biases of single-centre studies. While we cannot exclude the possibility that there may have been a few cases that were diagnosed but not reported, we have no reason to suspect substantial under-ascertainment or bias in reporting. This study therefore represents a true snapshot of the current number of diagnosed cases in the UK pregnant population, with the caveat that only a small number of centres are actively screening for vasa praevia.

We used a robust case definition in order to accurately capture clinically diagnosed and confirmed cases. However, this strict definition was a potential weakness, as demonstrated by the 10 antenatally suspected cases who could not be included as there was no evidence of examination (histopathological or not) of the placenta after birth. Photographic documentation during caesarean birth could have been considered as an alternative means of confirming the diagnosis. Although we had multiple reporters in each hospital, we cannot be certain all cases were identified. In particular, the diagnosis may never have been considered as there is a possibility that ruptured vasa praevia mimics other conditions (e.g., abruption) and there is no “gold” standard to confirm the diagnosis after birth if the vessels are not ruptured.

There are guidelines about the diagnosis and management of vasa praevia in the UK, USA, Canada and Australia highlighting the importance of the condition. These were compared in a recent publication ^20^. All guidelines agree that colour and pulsed Doppler transvaginal ultrasound should be used in mid-trimester to diagnose the condition, but third trimester confirmation is also needed. Universal screening is not recommended but rather targeted screening for women with risk factors, such as low placenta, is advocated in the U.S.A, Canada and Australia ^21– 23^. The UK’s Royal College of Obstetricians and Gynaecologists and National Screening Committee advise that further research into the balance of benefit and harm of targeted screening is needed before a recommendation can be made ^6, 24^. Antenatal steroids are to be considered from 28–32 weeks and hospitalisation from 30–32 weeks. All four guidelines agree the optimal timing of birth is unknown, but they recommend birth by caesarean section at 34–37 weeks. A recent expert consensus statement recommended caesarean birth at 35–37 weeks, but advocated second-trimester screening as routine for all pregnancies, via transabdominal ultrasound with colour Doppler sweep over the cervix, followed by third-trimester confirmation of the diagnosis by transvaginal ultrasound ^9^.

Our study demonstrates that vasa praevia diagnosis in the UK is less common than anticipated, with a lower estimated incidence of vasa praevia than found in previous studies ^1, 10, 13^. Even if the 10 planned caesarean sections with no postnatal confirmation were included, the estimated incidence would still be relatively low at 7.94 per 100,000 maternities (1 case for every 12,593 maternities). Although our strict case definition may be partially responsible, the AMOSS study in Australia demonstrated about 2–3 times higher incidence (21 per 100,000) using an almost identical case definition. However, in the Australian study, 92% of cases were identified antenatally with only 5 cases being identified peripartum. Peripartum cases might be considered to represent the true incidence of ruptured vasa praevia; the incidence of peripartum vasa praevia in Australia was 1.7 per 100,000, and in the UK it was 3 per 100,000. The differing thresholds and screening protocols for detecting vasa praevia antenatally are likely to explain these apparent differences in the overall reported incidence between the two countries.

Other possible reasons include cases not being reported or not recognised during the study period; women with undiagnosed vasa praevia having pre-labour caesarean section for other indications (especially low lying placenta); and some cases with poor outcome could have been attributed to other causes, such as abruption, or ruptured fetal vessels on placental histopathology being attributed to “snapped” cord during continuous cord traction at delivery, which is more likely to occur with velamentous cord insertion.

Our study found very different outcomes in those cases that were identified antenatally compared with those diagnosed intrapartum or postnatally. In cases not diagnosed antenatally there was an almost 40% perinatal mortality. This high perinatal mortality is remarkably similar to that observed in a retrospective study (44%) ^3^ and in the prospective AMOSS study (40%) ^1^ and confirms the high fatality rate of vasa praevia when it is not suspected or diagnosed before labour. Our study also found that almost half of the survivors in cases with no antenatal diagnosis had hypoxic ischaemic encephalopathy, although the grade and consequences of this are unknown; in contrast, none of the infants in cases with antenatal diagnosis had hypoxic ischaemic encephalopathy. This is in keeping with the findings of a recent systematic review, which found that 58% of surviving neonates in cases of vasa praevia without antenatal diagnosis displayed hypoxic morbidity, compared with only 2.7% in cases with antenatal diagnosis ^4^.

Most previous studies ^25^ demonstrated the presence of risk factors in most vasa praevia cases; in our study all cases had at least one risk factor. This might suggest that targeted screening for cases with risk factors, particularly low placenta and velamentous cord insertion, might be useful as one or both of these were present in almost half of the cases in our study. This would be consistent with current US, Canadian and Australian guidelines, all of which recommend targeted screening ^20^.

Ten maternity units declared they had a formal screening programme for vasa praevia. Of these, only one centre reported any cases of antenatally detected vasa praevia during the study period. Table 5 illustrates that the number of reported cases of vasa praevia was substantially higher if there was antenatal screening. Large, well-designed prospective screening studies may elucidate this observation further; our study was not designed to evaluate screening efficiency or accuracy. A historical cohort study of a screening program in a single UK institution reported 100% sensitivity and 99.78% specificity, but a perinatal mortality of 5% (one death in 19 confirmed cases of vasa praevia) ^15^.

Evaluation of accuracy of screening methods for vasa praevia is difficult because of the absence of a diagnostic “gold standard”. Once the placenta is delivered, velamentous cord insertion or bilobed placenta can be confirmed but it is impossible to know what the proximity of the vasa praevia to the cervix was. Inspection during the caesarean section is often difficult because of bleeding from the uterine incision and the placental bed. In addition, there is no clear definition of vasa praevia as there is no evidence about a “safe” distance from the internal os. A distance of 2 cm between the fetal vessels and the internal os has been proposed ^21, 26^ but it is not based on robust evidence. It is likely that many clinicians would find it difficult to recommend expectant management and normal birth to a woman with fetal vessels at e.g. 2.3 cm from the cervix. Indeed, a recent expert consensus process failed to reach agreement on the fetal vessel-internal os distance that should be used to define vasa praevia, but concluded that it should not be limited to 2 cm ^9^, and a recently registered clinical trial of fetoscopic laser photocoagulation in management of vasa praevia ( https://clinicaltrials.gov/study/NCT06290232) will use a 5-cm threshold for diagnosis. A UK single-institution study of a two-stage screening strategy for vasa praevia used a diagnostic threshold of 5 cm and estimated an incidence of 8 per 10,000 singleton pregnancies ^14^. If reporting centres in our study were using more restrictive criteria, this may go some way to explaining the much lower incidence that we observed.

The Royal College of Obstetricians and Gynaecologists ^6^ recommends consideration of prophylactic hospitalisation for confirmed vasa praevia after 30–32 weeks. Our study showed a wide variation in practice with most women with an antenatal diagnosis of vasa praevia being admitted after 30 weeks, at a median gestation of 32–33 weeks. Although most women spent up to 18 days in hospital in the antenatal period, there were 2 women who were admitted for over 2 months. The 4 women who did not have antenatal hospital admission had later planned caesarean sections at 38–39 weeks, suggesting the absence of any episodes of antepartum bleeding, no risk factors for early labour or even perhaps maternal preference.

Our data showed that most caesarean sections for the antenatally diagnosed cases were scheduled for 36–37 weeks and some up to 39 weeks. In about one third of these cases the caesarean section had to be brought forward, usually because of episodes of bleeding or threatened preterm labour. Only 2 caesarean sections (7%) were performed before 34 weeks. Within the antenatally diagnosed group there were only 2 cases of respiratory distress syndrome but 10 (36%) babies were admitted to a neonatal unit. Given the recently recognised additional risks of late preterm birth ^5, 27^, it would seem sensible to aim for delivery at 36–37 weeks and perform earlier caesarean sections for cases with episodes of vaginal bleeding or other risk factors. Clinicians can also consider using cervical length and/or fetal fibronectin or other cervicovaginal biochemical swab tests to stratify further the risk of preterm labour before 35 weeks of gestation although there is no current evidence to support this approach ^28^. Of course, maternal anxiety cannot be underestimated in these circumstances ^29^ and can be a significant factor in birth timing.

## Conclusion

This prospective national study estimated a lower than anticipated incidence of vasa praevia, and a very low population incidence of poor perinatal outcome after vasa praevia. However, peripartum diagnosis was associated with an almost 40% perinatal mortality and about 50% risk of HIE in the surviving neonates. Antenatal diagnosis led to planned caesarean section for the majority of infants at 36–37 weeks with no observed deaths or cases of HIE, but some infants had respiratory morbidity. Our study could not address screening efficacy or the optimal screening method, but showed that a screening programme was associated with an increased number of vasa praevia diagnoses and no perinatal deaths, although the difference on the latter metric between maternity units with and without a screening program did not reach statistical significance.

## Data Availability

Data cannot be shared because of confidentiality issues and potential identifiability of sensitive data as identified in the Research Ethics Committee approval. Requests to access the data can be made by contacting the National Perinatal Epidemiology Unit data access committee via general@npeu.ox.ac.uk. The estimated response time for requests is 4 weeks. Data sharing outside the UK or the European Union may require consultation with the UK Health Research Authority. For more information, please refer to the National Perinatal Epidemiology Unit Data Sharing Policy available at: https://www.npeu.ox.ac.uk/assets/downloads/npeu/policies/Data_Sharing_Policy.pdf
