# Supplementary material for: Incidence and outcomes of vasa praevia in the United Kingdom
Source: NIHR Open Res. 2024 Dec 5;4:49. Originally published 2024 Sep 3. [Version 2] doi: 10.3310/nihropenres.13696.2 (PMC11443191; doi:10.3310/nihropenres.13696.2)
Supplement: Peer review history

# Reviewer report: Reviewer response for version 2

**DOI:** 10.3310/nihropenres.14995.r34164

**Published:** 2025-01-17

**Authors:** Mena Abdalla

**Affiliations:** 1 King’s College Hospital NHS Foundation Trust, London, UK

This study provides valuable insights into the incidence and outcomes of vasa praevia using prospective, population-based data from the UK Obstetric Surveillance System (UKOSS). The study highlights a high perinatal mortality rate (37.5%) in undiagnosed cases and the absence of perinatal deaths in antenatally diagnosed cases, emphasizing the importance of early detection.

**Major Concerns:**

 1-      The study states that 10 hospitals (5%) had a formal screening program, but it is unclear whether these programs were universal or risk-based. Given that screening approaches can significantly influence detection rates, the authors should:

-  Specify how these screening programs operated
- Discuss whether screening was transvaginal or transabdominal ultrasound-based
- Clarify how screening influenced antenatal detection rates

 

 2-      A significant number of suspected cases (22 out of 73, or 30%) were excluded due to lack of histopathological confirmation. Given that: Vasa praevia is difficult to confirm postpartum, even with placental examination.

 The authors should:

- Provide more details on why these cases were excluded
- Consider whether photographic or intraoperative documentation could be used as an alternative to histopathology

3-      The rising use of ART is an important contributor to vasa praevia risk, yet it is only briefly mentioned. Given that ART pregnancies have a 10-20x higher risk of vasa praevia, the discussion should:

- Expand on how increasing ART usage may impact future incidence rates
- Discuss whether targeted screening for ART pregnancies should be considered

 

 4-      While the antenatal diagnosis group had no perinatal deaths, the conclusion that screening reduces mortality is not fully supported because:

 ·       Only 10 hospitals had screening programs, and 9 reported no cases

 ·       The statistical significance of screening impact is unclear

 ·       Some pregnancies diagnosed antenatally may never have resulted in adverse outcomes, even without screening

 The authors should:

-  Clarify that the impact of screening on perinatal outcomes remains uncertain
-  Emphasize the need for larger, well-designed studies to evaluate screening efficacy

 

**Minor Concerns:**

 In the discussion; discuss the potential role of cervical length measurement in guiding timing of delivery?

 Add a section for limitations.

 Fix minor spelling and grammatical errors ("form" instead of "from" in Methods section of the abstract)

Is the work clearly and accurately presented and does it cite the current literature?

Yes

If applicable, is the statistical analysis and its interpretation appropriate?

Yes

Are all the source data underlying the results available to ensure full reproducibility?

Yes

Is the study design appropriate and is the work technically sound?

Yes

Are the conclusions drawn adequately supported by the results?

Yes

Are sufficient details of methods and analysis provided to allow replication by others?

Yes

Reviewer Expertise:

Obstetrics and Gynaecology, fetal medicine, high risk obstetrics, maternal medicine.

I confirm that I have read this submission and believe that I have an appropriate level of expertise to confirm that it is of an acceptable scientific standard, however I have significant reservations, as outlined above.

# Reviewer report: Reviewer response for version 2

**DOI:** 10.3310/nihropenres.14995.r33986

**Published:** 2024-12-19

**Authors:** Alessandro Libretti

**Affiliations:** 1 University Hospital Maggiore della Carità, Novara, Italy

Review of the Article: *Incidence and Outcomes of Vasa Praevia in the United Kingdom*

 This study provides valuable insights into the incidence and outcomes of vasa praevia using robust, population-based data from UKOSS. It highlights the significant perinatal mortality (37.5%) in undiagnosed cases and the absence of deaths with antenatal diagnosis, emphasizing the importance of early detection.

 Strengths:

- **Study Design**: National, prospective data with rigorous case definitions ensure reliable results.
- **Clinical Relevance**: Clear evidence of improved outcomes with antenatal diagnosis supports the value of targeted screening.

Areas for Improvement:

1. **Low Incidence**: The reported rate (6.64 per 100,000) is lower than international studies. The authors should expand on potential factors, including stringent criteria and underreporting.
2. **Screening Programs**: More details are needed on the nature of screening protocols (universal vs. risk-based) in the few units reporting success.
3. **Broader Context**: Discuss the rising use of ART and its impact on future incidence and screening needs.

Conclusion:

 This study underscores the critical role of antenatal diagnosis but raises questions about the low incidence and limited screening uptake. Clarifying screening approaches and international comparisons would enhance its clinical impact.

Is the work clearly and accurately presented and does it cite the current literature?

Yes

If applicable, is the statistical analysis and its interpretation appropriate?

Yes

Are all the source data underlying the results available to ensure full reproducibility?

Yes

Is the study design appropriate and is the work technically sound?

Yes

Are the conclusions drawn adequately supported by the results?

Yes

Are sufficient details of methods and analysis provided to allow replication by others?

Yes

Reviewer Expertise:

Fetal medicine, ultrasound, prenatal diagnosis, obstetrics

I confirm that I have read this submission and believe that I have an appropriate level of expertise to confirm that it is of an acceptable scientific standard.

# Reviewer report: Reviewer response for version 1

**DOI:** 10.3310/nihropenres.14872.r32820

**Published:** 2024-09-30

**Authors:** Cristina Trilla

**Affiliations:** 1 Department of Obstetrics and Gynaecology, Hospital de la Santa Creu i Sant Pau, Autonomous University of Barcelona, Barcelona, Catalonia, Spain

Attilakos and co-authors present a comprehensive study on the incidence of vasa previa in the UK. From their results, it is clear that a prenatal diagnosis is critical for a good neonatal oucome. The authors used a prospective, population-based study design using the UK Obstetric Surveillance System (UKOSS), which should ensure a correct national representation. However, I am concerned by the very low incidence of vasa previa the authors report and by the potential impact this may have on local policies regarding screening and management of the condition. This is why I would recommend a more cautious discussion on their findings.

 My main concerns are presented below:

  

1. Methods: the authors describe a screening program for vasa previa in some of the institutions involved in this survey. Please, provide more accurate information on these screening programs (universal? Risk-based?), as this could significantly affect prenatal diagnosis.
2. The definition criteria and confirmation criteria for vasa previa was very strict. Sometimes vasa previa is difficult to diagnose post-partum, even with a careful examination of the placenta and the cord. This could explain the unexpected low rate of vasa previa in the study. The authors have included this as a limitation of the study, but I would ask the authors to elaborate more regarding the added value of this research considering this limitation. I understand that the aim of the study was to provide accurate information on vasa previa incidence in the UK, but these results are not concordant with previous research on the topic in other countries, and there is no clear explanation for this very low incidence in the UK. In any case, vasa previa incidence should be increasing, mainly due to the increase of ART among other risk factors. This should be mentioned and discussed more clearly in the manuscript, as such data may impact management policies for the condition if the incidence is really this low.
3. Many cases were excluded (22 out of 73, which represents 30% of the suspected cases). I do not clearly understand the exclusion criteria for these, and I would suggest revising this, as I believe it has greatly affected the final numbers. Examination of the placenta was apparently a requirement for diagnosis, but how often is this really performed?
4. Please, provide accurate definition for urgency categories for CS in methods.
5. If I understand correctly, 2015 maternity centers participated in the study. As such, many maternity centers did not report any cases of vasa previa. Did the number of deliveries per maternity center correlate with the number of vasa previa diagnosed (either antenatally or postnatally)? Please, provide more data regarding the number the deliveries per center and the number of cases diagnosed.
6. I believe one of the main conclusions should be that a more clear definition of vasa previa and its diagnosis is needed, rather than that its incidence in the UK is lower than in other countries.

My minor concerns are presented below:

1. Introduction: the authors are only referring to vasa previa type 1 and 2, but a third type has been described. This should be mentioned in the introduction
2. Underreporting of vasa previa: They state there is no reason to believe there were any underreported cases, but this is impossible to ascertain and should be listed as a limitation for the study.
3. Minor spelling error in the Methods section of the abstract: “form” should be spelled “from”. I did not find any other spelling concerns.

Is the work clearly and accurately presented and does it cite the current literature?

Yes

If applicable, is the statistical analysis and its interpretation appropriate?

Yes

Are all the source data underlying the results available to ensure full reproducibility?

Yes

Is the study design appropriate and is the work technically sound?

Partly

Are the conclusions drawn adequately supported by the results?

Partly

Are sufficient details of methods and analysis provided to allow replication by others?

Yes

Reviewer Expertise:

Prenatal diagnosis, placenta previa and vasa previa. Pregnancy loss.

I confirm that I have read this submission and believe that I have an appropriate level of expertise to confirm that it is of an acceptable scientific standard, however I have significant reservations, as outlined above.

# Response

**Published:** 2024-11-26

**Authors:** Ruth Tunn

**Affiliations:** NPEU, University of Oxford, Oxford, England, UK

***Reviewer Comments:***

*Methods: the authors describe a screening program for vasa previa in some of the institutions involved in this survey. Please, provide more accurate information on these screening programs (universal? Risk-based?), as this could significantly affect prenatal diagnosis.*

**Author Response: **Unfortunately, we do not have further details of the screening protocols used at the centres in question at the time of the study. UKOSS reporting operates on a volunteer basis, and to avoid overburdening reporters and maximise return rates, we limit the amount of information requested. This study did not have evaluation of screening as a main objective, so we limited data collection about screening programmes to the simple yes/no question described in the methods. We present data about screening programmes in relation to diagnosis in Table 5 to encourage and inform future research into this important issue, but do not seek to draw recommendations from the limited information collected.

 -------------

***Reviewer Comments:***

*The definition criteria and confirmation criteria for vasa previa was very strict. Sometimes vasa previa is difficult to diagnose post-partum, even with a careful examination of the placenta and the cord. This could explain the unexpected low rate of vasa previa in the study. The authors have included this as a limitation of the study, but I would ask the authors to elaborate more regarding the added value of this research considering this limitation. I understand that the aim of the study was to provide accurate information on vasa previa incidence in the UK, but these results are not concordant with previous research on the topic in other countries, and there is no clear explanation for this very low incidence in the UK. In any case, vasa previa incidence should be increasing, mainly due to the increase of ART among other risk factors. This should be mentioned and discussed more clearly in the manuscript, as such data may impact management policies for the condition if the incidence is really this low.*   

**Author Response: **As we note in the discussion, the clearly defined criteria we used meant that suspected cases diagnosed following screening could not be included if there was no postnatal examination of the placenta and membranes to confirm an abnormal vessel. As we describe in our response to reviewer 2 concerning incidence, the important point to note is the difference between diagnosed VP based on screening, and diagnosed VP based on adverse outcome. As few hospitals in the UK have VP screening programmes, the estimate of incidence is more reflective of the latter, which may partly explain why it is lower than some other estimates, although notably similar to studies using similar methodology (Sullivan EA, Javid N, Duncombe G, Li Z, Safi N, Cincotta R, et al. Vasa previa diagnosis, clinical practice, and outcomes in Australia. Obstetrics & Gynecology. 2017;130(3):591-8). Both our study and the AMOSS study are nationwide population studies in contrast to single centre VP screening studies, which report higher incidence.

 -------------

***Reviewer Comments:***

*Many cases were excluded (22 out of 73, which represents 30% of the suspected cases). I do not clearly understand the exclusion criteria for these, and I would suggest revising this, as I believe it has greatly affected the final numbers. Examination of the placenta was apparently a requirement for diagnosis, but how often is this really performed?*

**Author Response: **UKOSS collects data prospectively, meaning clinicians are asked to be alert to cases of the specified conditions being studied at any given time and to report them. This potentially increased the frequency of confirmatory examination of the placenta beyond that seen in normal clinical practice, helping to ensure cases were captured. As indicated in Table 3, the placenta was examined in 100% of antenatally diagnosed cases and 91% of peripartum-diagnosed cases; it was sent to pathology for further examination in 32% of antenatally diagnosed cases and 65% of peripartum-diagnosed cases.

 We provide additional information about the excluded “cases” in Figure 1. Of the 22 reports of apparent cases that were excluded, one fell outside the study period and seven were found when reviewed to not meet the case definition (two were cases of cord prolapse, two were placenta praevia, and three were other non-vasa praevia reports). It would therefore be inappropriate to consider these in calculation of vasa praevia incidence.

 Of the remaining fourteen reports received but not confirmed as vasa praevia, four were of women who had antepartum haemorrhage, fetal anaemia, or other poor outcomes. These outcomes are sometimes associated with vasa praevia but could be due to a range of other causes, and there was no indication that vasa praevia was suspected antenatally. Because UKOSS reporters are asked to identify and report all cases of the condition in question, they will sometimes err on the side of caution and include reports of observations that might be of interest, “just in case”, to allow the UKOSS researchers on the specific study to make the final adjudication. However, there was little evidence in these cases to suspect vasa praevia specifically.

 The remaining ten excluded reports were of women in whom vasa praevia was suspected antenatally and who underwent caesarean section indicated by vasa praevia, but there was no documented confirmation of the condition. We could therefore not include these under our case definition in the primary estimate of incidence; however, we do discuss the possible impact of their exclusion in the Discussion: “ *Even if the 10 planned caesarean sections with no postnatal confirmation were included, the estimated incidence would still be relatively low at 7.94 per 100,000 maternities (1 case for every 12,593 maternities)*.”

 We have expanded on the reasons for exclusion in Figure 1 and changed the terminology of unconfirmed “cases” to “reports” in the text for clarity.

 -------------

***Reviewer Comments:***

*Please, provide accurate definition for urgency categories for CS in methods.*

**Author Response: **We have added a link to the full data collection form in the Methods, which includes the classification of urgency as provided to the reporting clinicians, in the “Definitions” section. We have also provided the definitions in the footnote to Table 3.

 -------------

***Reviewer Comments:***

*If I understand correctly, 2015 maternity centers participated in the study. As such, many maternity centers did not report any cases of vasa previa. Did the number of deliveries per maternity center correlate with the number of vasa previa diagnosed (either antenatally or postnatally)? Please, provide more data regarding the number the deliveries per center and the number of cases diagnosed.*

**Author Response: **We apologise for any confusion. In total, there are 193 consultant-led maternity units in the UK, all of which participate in UKOSS. The 2015 annual statistics for the constituent countries of the UK were used as the denominator for the incidence calculation, rather than data on the number of deliveries per individual maternity centre being collected in this study. Examining case distribution by centre would risk identification of sensitive information given the small numbers involved, and would be unlikely to be informative given the very limited statistical power of this analysis.

 -------------

***Reviewer Comments:***

*I believe one of the main conclusions should be that a more clear definition of vasa previa and its diagnosis is needed, rather than that its incidence in the UK is lower than in other countries.*

**Author Response: **We state in the Discussion that differing thresholds and screening protocols for detecting vasa praevia antenatally are likely to explain the apparent differences in the overall reported incidence between countries. For example, a variable distance of 2-5 cm from the internal cervical os is used to diagnose vasa praevia in ultrasound screening. We also discuss other possible reasons for the apparent differences between countries (under-diagnosis, underreporting, mis-diagnosis).

 -------------

***Reviewer Comments:***

*My minor concerns are presented below:*

*Introduction: the authors are only referring to vasa previa type 1 and 2, but a third type has been described. This should be mentioned in the introduction*

**Author Response: **When this study was designed and conducted (2014–2015), type III vasa praevia had not been described as a distinct subtype. It was therefore not explicitly considered as a sub-type in the case definition or eligibility criteria. However, cases of VP of this type would have been captured by the case definition.

 We have added a mention of type III vasa praevia to the Introduction.

 -------------

***Reviewer Comments:***

*Underreporting of vasa previa: They state there is no reason to believe there were any underreported cases, but this is impossible to ascertain and should be listed as a limitation for the study.*

**Author Response: **We state in the Discussion section: “Although we had multiple reporters in each hospital, we cannot be certain all cases were identified. In particular, the diagnosis may never have been considered as there is a possibility that ruptured vasa praevia mimics other conditions (e.g., abruption) and there is no “gold” standard to confirm the diagnosis after birth if the vessels are not ruptured.”

 We also mention that misattribution of cases to other causes, and cases not being reported or not recognised during the study period, are possible reasons for the lower-than-expected estimate of incidence.

 As mentioned above, the important point to note is the difference between diagnosed vasa praevia based on screening, and diagnosed vasa praevia based on adverse outcome. As few hospitals in the UK have vasa praevia screening programmes, the estimate of incidence is more reflective of the latter.

 -------------

***Reviewer Comments:***

*Minor spelling error in the Methods section of the abstract: “form” should be spelled “from”. I did not find any other spelling concerns.*

**Author Response: **Thank you, we have corrected this error.

# Reviewer report: Reviewer response for version 1

**DOI:** 10.3310/nihropenres.14872.r32941

**Published:** 2024-09-30

**Authors:** Paolo Ivo Cavoretto

**Affiliations:** 1 Vita-Salute San Raffaele University, Milan, Italy

This is a very good study from a known research group in the UK, presenting a compelling study topic and aim, adequate rationale and methodology and acceptable conclusions. I have few remarks and recommendations to improve this interesting paper:

 1. Type III vasa previa was not considered enough by the authors and this in an important reticence to be corrected. Type III vasa previa is a rare form of VP, not necessarily associated with other placental or vascular anomalies, in which aberrant vessels run from the placenta to the amniotic membranes, near the internal cervical os, before returning to the placenta.  It was object of a recent systematic review published in UOG that should be considered by the authors (ref 1).

 Were type III VP accounted for? Were they excluded from the study? This is very interesting to be added for readers. A wide dissemination on knowledge around type III vasa previa is required as this is not yet a well-known topic. 

 2. The authors state that in 10 cases "no documentation of the placenta being examined or sent to histopathology after birth, so there was no confirmation of vasa praevia". However I am afraid that anatomopatological diagnosis simply cannot  rule out or rule in vasa previa, as the relationship with the uterine structures is only available as an intraoperative surgical macroscopic diagnosis. This diagnosis can be documented with pictures and videos or with clinical diagnosis during delivery Anatomopatologists can only diagnose velamentous cord insertion, bilobate/succenturiate or type III extraplacental aberrant vessels as well as placental structural or morpholigcal aberrations associated (but not patognominic) to vasa previa. This comment is not against the authors findings but instead it may be useful to reinforce their message (there is an extent of wrong prenatal diagnosis but this risk in expert hands is very low).

 3. Methods. Among risk factors for VP multiple pregnancies should be included.

 4. The issue of cervical screening for cervical lenght, to be used to screen also for vasa previa screening may be considered and mentioned with pros and cons.

 I congratulate with the authors for this brilliant work.

Is the work clearly and accurately presented and does it cite the current literature?

Partly

If applicable, is the statistical analysis and its interpretation appropriate?

Yes

Are all the source data underlying the results available to ensure full reproducibility?

Yes

Is the study design appropriate and is the work technically sound?

Yes

Are the conclusions drawn adequately supported by the results?

Yes

Are sufficient details of methods and analysis provided to allow replication by others?

Yes

Reviewer Expertise:

I am expert in maternal fetal medicine and obstetrics and I did previous research on the topic. I feel comfortable in providing a review on the paper. in object.

I confirm that I have read this submission and believe that I have an appropriate level of expertise to confirm that it is of an acceptable scientific standard.

## References

[1] : Prenatal diagnosis and postnatal outcome of Type-III vasa previa: systematic review of literature. *Ultrasound Obstet Gynecol* .2024;63(1) : 10.1002/uog.26315 24-33 10.1002/uog.26315 37470694

# Response

**Published:** 2024-11-26

**Authors:** Ruth Tunn

**Affiliations:** NPEU, University of Oxford, Oxford, England, UK

*1. Type III vasa previa was not considered enough by the authors and this in an important reticence to be corrected. Type III vasa previa is a rare form of VP, not necessarily associated with other placental or vascular anomalies, in which aberrant vessels run from the placenta to the amniotic membranes, near the internal cervical os, before returning to the placenta.  It was object of a recent systematic review published in UOG that should be considered by the authors (ref 1).*

*Were type III VP accounted for? Were they excluded from the study? This is very interesting to be added for readers. A wide dissemination on knowledge around type III vasa previa is required as this is not yet a well-known topic.*

 Response: When this study was designed and conducted (2014–2015), type III vasa praevia had not been described as a distinct subtype. It was therefore not explicitly considered as a sub-type in the case definition or eligibility criteria. However, cases of VP of this type would have been captured by the case definition. Indeed, in some scientific presentations before 2014, the term "type III vasa praevia” was used to describe vasa praevia of a second twin when its membranes were presenting after the birth of the first twin. This type of vasa praevia would also have been captured by the case definition.

 We have added a mention of type III vasa praevia to the Introduction.

*2. The authors state that in 10 cases "no documentation of the placenta being examined or sent to histopathology after birth, so there was no confirmation of vasa praevia". However I am afraid that anatomopatological diagnosis simply cannot  rule out or rule in vasa previa, as the relationship with the uterine structures is only available as an intraoperative surgical macroscopic diagnosis. This diagnosis can be documented with pictures and videos or with clinical diagnosis during delivery Anatomopatologists can only diagnose velamentous cord insertion, bilobate/succenturiate or type III extraplacental aberrant vessels as well as placental structural or morpholigcal aberrations associated (but not patognominic) to vasa previa. This comment is not against the authors findings but instead it may be useful to reinforce their message (there is an extent of wrong prenatal diagnosis but this risk in expert hands is very low).*

 Response: Thank you, we have added to the discussion the consideration of the potential value of intra-operative photographic documentation.

*3. Methods. Among risk factors for VP multiple pregnancies should be included.*

 Response: Data were collected on multiple pregnancies, but numbers were too small to be meaningfully analysed as only one woman with a multiple pregnancy was affected. We have added a link to the full data collection form for the study. Furthermore, in the meta-analysis by Ruiter et al, multiple pregnancy was not found to be a statistically significant risk factor.

*4. The issue of cervical screening for cervical length, to be used to screen also for vasa previa screening may be considered and mentioned with pros and cons.*

 Response: As indicated in Table 3, cervical length was measured in 36% of antenatally diagnosed cases but used in the decision to admit the woman to hospital in only 11%. Cervical length was not measured in any of the peripartum-diagnosed women.

# Reviewer report: Reviewer response for version 1

**DOI:** 10.3310/nihropenres.14872.r32819

**Published:** 2024-09-27

**Authors:** Ramesha Papanna, Neha Agarwal

**Affiliations:** 1 Department of Obstetrics, Gynecology and Reproductive Sciences, McGovern Medical School, The University of Texas, Houston, TX, USA; 2 Obstetrics and Gynecology, The University of Texas Health Science Center at Houston John P and Katherine G McGovern Medical School, Houston, Texas, USA

Attilakos et al are presenting a population based prospective study of vasa previa in UK to demonstrate registered incidence of vasa previa and its outcomes. The study confirms the well-known knowledge of antenatal diagnosis is critical in reducing perinatal mortality and morbidity of vasa previa. It also reflects upon the effect of lack of screening policy for vasa previa and its impact on the perinatal outcomes.

 Below are the major concerns of the article:

1. Limited Scope of Screening Analysis:  While the article mentions that only 5% of obstetric units in the UK had screening programs for vasa previa, it does not provide sufficient detail about the methods or criteria used for these screenings. Although there are significant differences in outcomes between hospitals with and without screening programs, the study lacks a detailed exploration of the specific screening strategies that could help reduce perinatal mortality and morbidity. A more in-depth investigation into the screening protocols used by the 10 units with programs would be beneficial. This additional detail could help inform future recommendations for the implementation of nationwide screening programs.
2. Exclusion of cases in antepartum vs postpartum: Need this distinction made to help improve the clarity of case selection. Of those 22 excluded cases are from antenatal diagnosis group, due to lack of sufficient details, it would help to make a better determination of the incidence.
3. Underestimation of the vasa previa. As authors acknowledge in the weakness of the study, there is high probability of underestimation of vasa previa due to nature of reporting in this large-scale study. There is lack of rigorous methods to determine the true incidence of vasa previa. Consider avoiding “incidence” in the title, objective, and use “estimates” .
4. Confirmation of vasa previa: Lack of details in the diagnosis of vasa previa including exposed fetal vessels on clinical examination. How often is clinical examination performed of the placenta? Is this universal? Are there standard protocols that require post-delivery placental evaluation to ensure optimal case capture?   
5. Confirmation of pathologic examination of the placenta”  How many in this cohort? How can you confirm that without information on placental orientation within the uterine environment?
6. It is recommended that for the authors to estimates of effect of universal screening for vasa previa similar to 6% of hospitals on the obstetrical outcomes- number of c-sections to prevent one perinatal death and hypoxic ischemic encephalopathy. Does this justify the national guidelines for universal screening for vasa previa.

 Below are the minor concerns of the article:

1. In the Methods section of the abstract, there is a spelling error in line 3. The word "form" should be corrected to "from."
2. 48% of the cases of peripartum identification had an antepartum admission. What were the indications for admission? Was this preterm labor? Vaginal bleeding? Unrelated medical indications (e.g., preeclampsia)?
3. Irrespective of universal screening recommendation, are there recommendations for screening among those with risk factors considering 43% of the cases with peripartum identification had low-lying placenta and/or velamentous cord insertion and 100% had at least 1 risk factor.
4. In the 7 ^th^ paragraph of the Results section, why term “no antenatal diagnosis” rather than “peripartum” as is otherwise used throughout the article?

Is the work clearly and accurately presented and does it cite the current literature?

Yes

If applicable, is the statistical analysis and its interpretation appropriate?

Partly

Are all the source data underlying the results available to ensure full reproducibility?

Yes

Is the study design appropriate and is the work technically sound?

No

Are the conclusions drawn adequately supported by the results?

Partly

Are sufficient details of methods and analysis provided to allow replication by others?

Yes

Reviewer Expertise:

Vasa previa, placenta accreta and fetal surgery

We confirm that we have read this submission and believe that we have an appropriate level of expertise to state that we do not consider it to be of an acceptable scientific standard, for reasons outlined above.

# Response

**Published:** 2024-11-26

**Authors:** Ruth Tunn

**Affiliations:** NPEU, University of Oxford, Oxford, England, UK

*1. Limited Scope of Screening Analysis:  While the article mentions that only 5% of obstetric units in the UK had screening programs for vasa previa, it does not provide sufficient detail about the methods or criteria used for these screenings. Although there are significant differences in outcomes between hospitals with and without screening programs, the study lacks a detailed exploration of the specific screening strategies that could help reduce perinatal mortality and morbidity. A more in-depth investigation into the screening protocols used by the 10 units with programs would be beneficial. This additional detail could help inform future recommendations for the implementation of nationwide screening programs.*

 Response: Unfortunately, we do not have further details of the screening protocols used at the centres in question at the time of the study. UKOSS reporting operates on a volunteer basis, and to avoid overburdening reporters and maximise return rates, we limit the amount of information requested. This study did not have evaluation of screening as a main objective, so we limited data collection about screening programmes to the simple yes/no question described in the methods. We present data about screening programmes in relation to diagnosis in Table 5 to encourage and inform future research into this important issue, but do not seek to draw recommendations from the limited information collected.

*2. Exclusion of cases in antepartum vs postpartum: Need this distinction made to help improve the clarity of case selection. Of those 22 excluded cases are from antenatal diagnosis group, due to lack of sufficient details, it would help to make a better determination of the incidence.*

 Response: We provide additional information about the excluded “cases” in Figure 1. Of the 22 reports of apparent cases that were excluded, one fell outside the study period and seven were found when reviewed to not meet the case definition (two were cases of cord prolapse, two were placenta praevia, and three were other non-vasa praevia reports). It would therefore be inappropriate to consider these in calculation of vasa praevia incidence.

 Of the remaining fourteen reports received but not confirmed as vasa praevia, four were of women who had antepartum haemorrhage, fetal anaemia, or other poor outcomes. These outcomes are sometimes associated with vasa praevia but could be due to a range of other causes, and there was no indication that vasa praevia was suspected antenatally. Because UKOSS reporters are asked to identify and report all cases of the condition in question, they will sometimes err on the side of caution and include reports of observations that might be of interest, “just in case”, to allow the UKOSS researchers on the specific study to make the final adjudication. However, there was insufficient evidence in these cases to suspect vasa praevia specifically.

 The remaining ten excluded reports were of women in whom vasa praevia was suspected antenatally and who underwent planned caesarean section because of this suspicion, but there was no documented confirmation of the condition after women gave birth. We could therefore not include these under our case definition in the primary estimate of incidence; however, we do discuss the possible impact of their exclusion in the Discussion: “ *Even if the 10 planned caesarean sections with no postnatal confirmation were included, the estimated incidence would still be relatively low at 7.94 per 100,000 maternities (1 case for every 12,593 maternities)*.”

 We have expanded on the reasons for exclusion in Figure 1 and changed the terminology of unconfirmed “cases” to “reports” in the text for clarity.

*3. Underestimation of the vasa previa. As authors acknowledge in the weakness of the study, there is high probability of underestimation of vasa previa due to nature of reporting in this large-scale study. There is lack of rigorous methods to determine the true incidence of vasa previa. Consider avoiding “incidence” in the title, objective, and use “estimates” .*

 Response: We have retained the term “incidence”, as this was the metric that the study was designed to investigate, but have added “estimated” at relevant points in the text to reflect the uncertainty arising from the acknowledged limitations. The important point to note is the difference between diagnosed vasa praevia based on screening, and diagnosed vasa praevia based on adverse outcome. As few hospitals in the UK have vasa praevia screening programmes, the estimate of incidence is more reflective of the latter.

*4. Confirmation of vasa previa: Lack of details in the diagnosis of vasa previa including exposed fetal vessels on clinical examination. How often is clinical examination performed of the placenta? Is this universal? Are there standard protocols that require post-delivery placental evaluation to ensure optimal case capture?*

*5. Confirmation of pathologic examination of the placenta”  How many in this cohort? How can you confirm that without information on placental orientation within the uterine environment?*

 Response: Visual examination of all placentas post-birth is standard practice in the UK, to ensure completeness as well as to identify abnormal vessels. As indicated in Table 3, the placenta was examined in 100% of antenatally diagnosed cases (noting that this was required to establish the case definition) and 91% of peripartum-diagnosed cases; it was sent to pathology for further examination in 32% of antenatally diagnosed cases and 65% of peripartum-diagnosed cases. As the reviewer notes, in placentas of women who have had no suspicion of vasa praevia antenatally and no adverse outcome, the observation of fetal vessels in the membranes postnatally cannot be used to confirm vasa praevia as it would be unknown as to whether those vessels crossed the cervix, although the position of membrane rupture may be an indication. However, ruptured fetal vessels are readily detectable and, in women with antenatal suspicion in whom the orientation will have been confirmed on scan, fetal vessels running through the membranes can be confirmed postnatally.

*6. It is recommended that for the authors to estimates of effect of universal screening for vasa previa similar to 6% of hospitals on the obstetrical outcomes- number of c-sections to prevent one perinatal death and hypoxic ischemic encephalopathy. Does this justify the national guidelines for universal screening for vasa previa.*

 Response: We agree that this would be useful information to have. However, we do not think that our study design and the limited amount of data from the few centres with screening programmes can support such analyses or would yield robust conclusions/recommendations. The numbers involved are small, association between screening and antenatal diagnosis was not consistent across centres with programmes and, and as you identified in a previous comment, we have limited details of the exact composition of the screening programmes that the screening centres had in place at the time of the study, so a recommendation would lack necessary details of any advocated intervention.

 We say in the discussion, “Table 5 illustrates that the number of reported cases of vasa praevia was substantially higher if there was antenatal screening. Large, well-designed prospective screening studies may elucidate this observation further; our study was not designed to evaluate screening efficiency or accuracy”. We agree that future studies should investigate screening in more depth to be able to provide guidance on this issue.

*Below are the minor concerns of the article:*

*1. In the Methods section of the abstract, there is a spelling error in line 3. The word "form" should be corrected to "from."*

 Response: Thank you, we have corrected this error.

*2. 48% of the cases of peripartum identification had an antepartum admission. What were the indications for admission? Was this preterm labor? Vaginal bleeding? Unrelated medical indications (e.g., preeclampsia)?*

 Response: Of the 11 women in the peripartum diagnosis group admitted to hospital antenatally, three were admitted for reasons relating to the position or movement of the fetus; four for maternal indications unrelated to vasa praevia, such as elevated blood pressure; and two for antepartum haemorrhage that progressed to birth. Notably, two women were admitted for antepartum bleeding earlier in the pregnancy, which could indicate a missed opportunity for vasa praevia diagnosis.

 We have added this information to the manuscript.

*3. Irrespective of universal screening recommendation, are there recommendations for screening among those with risk factors considering 43% of the cases with peripartum identification had low-lying placenta and/or velamentous cord insertion and 100% had at least 1 risk factor.*

 Response: Our study suggests that targeted screening may be appropriate, although we are cautious about making firm recommendations on the basis of this evidence alone. National guidelines in the US, Canada and Australia all advocate targeted screening for women with risk factors for vasa praevia. We mentioned these guidelines in broader terms in the fourth paragraph of the Discussion, but have now emphasised the relevant recommendations again where we reflect on risk factors in the cases in our study, by adding the sentence in bold below:

 “Most previous studies (20) demonstrated the presence of risk factors in most vasa praevia cases; in our study all cases had at least one risk factor. This might suggest that targeted screening for cases with risk factors, particularly low placenta and velamentous cord insertion, might be useful as one or both of these were present in almost half of the cases in our study. **This would be consistent with current US, Canadian and Australian guidelines, all of which recommend targeted screening (19).**”

 19. Tsakiridis I, Mamopoulos A, Athanasiadis A, Dagklis T. Diagnosis and Management of Vasa previa: a comparison of 4 national guidelines. Obstetrical & Gynecological Survey. 2019;74(7):436-42.

*4. In the 7th paragraph of the Results section, why term “no antenatal diagnosis” rather than “peripartum” as is otherwise used throughout the article?*

 Response: Thank you for pointing this out, we have made the terminology consistent.

# Reviewer report: Reviewer response for version 1

**DOI:** 10.3310/nihropenres.14872.r32946

**Published:** 2024-09-18

**Authors:** Antonios Siargkas

**Affiliations:** 1 Aristotle University of Thessaloniki, Thessaloniki, Greece

"Incidence and Outcomes of Vasa Praevia in the United Kingdom" by Attilakos et al. is an excellent and comprehensive study that sheds light on the rare but critical obstetric condition of vasa praevia. The authors have executed a robust, prospective, population-based study using the UK Obstetric Surveillance System (UKOSS), ensuring a sound methodology and national representation. They provided a clear definition of vasa praevia and meticulously described the case reporting process by nominated clinicians across all UK hospitals with obstetrician-led maternity units. This approach ensured that specific risk factors—such as IVF conception, low-lying placenta, marginal or velamentous cord insertion, and bilobed or succenturiate lobed placentation—were thoroughly reported and analyzed.

 The statistical analysis was appropriately conducted and clearly presented, offering valuable insights into this rare pathological entity. The results were particularly interesting, revealing an incidence of diagnosed vasa praevia at 6.64 per 100,000 maternities, which is lower than anticipated. This equates to approximately one case per 15,062 maternities. The study highlighted significant differences in outcomes between antenatally diagnosed cases and those diagnosed peripartum.

 In the peripartum diagnosis group, there were four stillbirths and five neonatal deaths, resulting in a perinatal mortality rate of 37.5% for this group. The overall perinatal mortality rate was 17%. Moreover, almost 50% (seven out of 15) of the surviving babies in this group developed hypoxic ischaemic encephalopathy (HIE), and 46% required blood transfusions due to anaemia. In stark contrast, the antenatal diagnosis group reported no severe adverse outcomes, aside from a 36% admission rate to the neonatal intensive care unit (NICU), likely due to preterm delivery from planned caesarean sections.

 The discussion was highly informative and thoroughly covered the key points of the study. One of the main findings was the high perinatal mortality and morbidity associated with peripartum diagnosis of vasa praevia. The authors used a robust case definition to accurately capture clinically diagnosed and confirmed cases. However, they acknowledged that this strict definition might have been a potential weakness, as it excluded ten antenatally suspected cases without postnatal confirmation due to the lack of examination of the placenta after birth. This exclusion could have led to an underestimation of the true incidence, but the authors transparently discussed this limitation and provided relevant data.

 The study also reviewed relevant guidelines, noting that routine screening for vasa praevia is not currently advised. However, given that all cases in their study had at least one risk factor, the authors proposed that targeted screening for pregnancies with specific risk factors—particularly low-lying placenta and velamentous cord insertion—might be beneficial. This suggestion is compelling, as one or both of these risk factors were present in almost half of the cases. Such targeted screening could improve early detection without the drawbacks associated with universal screening.

 Interestingly, only ten hospitals (6% of respondents) had a formal screening programme for vasa praevia during the study period. One of these hospitals identified 25% of the antenatally diagnosed cases, indicating that formal screening programmes in high-risk populations can significantly enhance detection rates and improve outcomes.

 In conclusion, this study offers valuable insights into the incidence and outcomes of vasa praevia in the UK, highlighting the critical importance of antenatal diagnosis in improving perinatal outcomes. The authors have provided a well-structured and detailed analysis, with sound methodology and comprehensive reporting. Their proposal for targeted screening based on specific risk factors is particularly noteworthy and could inform future guidelines and clinical practice. Overall, this research represents a significant contribution to obstetric literature and has important implications for the management of pregnancies at risk of vasa praevia.

 Upon carefully reading the article, I found no mistakes or issues that require correction.

Is the work clearly and accurately presented and does it cite the current literature?

Yes

If applicable, is the statistical analysis and its interpretation appropriate?

Yes

Are all the source data underlying the results available to ensure full reproducibility?

Yes

Is the study design appropriate and is the work technically sound?

Yes

Are the conclusions drawn adequately supported by the results?

Yes

Are sufficient details of methods and analysis provided to allow replication by others?

Yes

Reviewer Expertise:

Maternal Fetal Medicine, placenta, umbilical cord, high risk pregnancies

I confirm that I have read this submission and believe that I have an appropriate level of expertise to confirm that it is of an acceptable scientific standard.

# Response

**Published:** 2024-11-26

**Authors:** Ruth Tunn

**Affiliations:** NPEU, University of Oxford, Oxford, England, UK

Thank you for your supportive comments.
